# Perceptions of youth-friendly sexual and reproductive health services in selected Higher and Tertiary Education Institutions of Zambia: A qualitative study on the perspectives of young people and healthcare providers

**DOI:** 10.1371/journal.pgph.0002650

**Published:** 2023-11-22

**Authors:** Choolwe Jacobs, Flata Mwale, Musonda Mubanga, Mwenya Kasonde, Alice Saili, Remmy Mukonka, Lenard Mumbi Mwilu, Margarate Nzala Munakampe

**Affiliations:** 1 Department of Epidemiology and Biostatistics, School of Public Health, University of Zambia, Lusaka, Zambia; 2 Women in Global Health, Lusaka, Zambia; 3 Department of Public Health, School of Medicine and Health Sciences, University of Lusaka, Lusaka, Zambia; 4 Liverpool School of Tropical Medicine, Liverpool, United Kingdom; 5 United Nations Educational, Scientific and Cultural Organization, Lusaka, Zambia; 6 Department of Health Policy and Management, School of Public Health, University of Zambia, Lusaka, Zambia; 7 Yakini Health Research Institute, Lusaka, Zambia; Tata Institute of Social Sciences, INDIA

## Abstract

The recognition of the need for Adolescent and Youth-Friendly Health Services (AYFHS) is growing. It is important to ensure the provision of high-quality sexual and reproductive health (SRH) services that cater to the unique needs of adolescents and young people (AYP). Adolescents and young people spend a significant amount of time in Higher and Tertiary Education Institutions (HTEIs) where adolescent friendly services are needed. However, there is limited evidence on the availability of sexual and reproductive health services for young people in HTEIs in Zambia. Using the Human Rights Based Approach to healthcare availability, accessibility, acceptability, and quality of care (AAAQ) framework, this study explores young people’s perceptions of youth-friendly sexual and reproductive health services in selected HTEIs in Zambia. Between March and June 2021, a qualitative case study was conducted in 12 selected HTEIs located in Lusaka, Central, and Copperbelt provinces of Zambia. The study employed In-depth Interviews (IDIs) and Focus Group Discussions (FGDs) with AYPs, as well as Key-informant Interviews (KIIs) with healthcare providers. The healthcare providers at health facility, district and provincial levels were interviewed to provide insights about the services provided in the HTEIs. A total of 112 interviews were conducted. Data analysis was performed using thematic analysis in NVivo version 11. In the study, young people reported the availability of primary health services like malaria, HIV, and pregnancy testing, as well as screening for STIs. However, their awareness of SRH services was limited. Contraception, STI testing and treatment, and HIV and pregnancy screening and testing were the most accessed services. Equipment and commodity shortages were common, hindering care provision. Young people found healthcare services in educational institutions inaccessible, with limited comprehensive care and understanding from providers. Services lacked tailoring for key populations and privacy/confidentiality. Health care providers also reported inadequate youth-friendly spaces, equipment, medication and trained workers which compromised the quality of care. Peer educators and provider training were seen as essential for improving accessibility and acceptability of services. The findings indicate significant barriers to the accessibility, availability, and acceptability of SRH services for AYP in HTEIs in Zambia. There is a pressing need to enhance AYSRH programming by increasing awareness of services and ensuring their availability and accessibility for young people. Sufficient funding for AYFHS can address challenges related to service quality, including inconsistent availability of medical equipment and supplies. Building the capacity of service providers can potentially enhance the uptake of services by AYP. It is crucial to target services to address the specific vulnerabilities of students with disabilities and key populations, aligning with the goal of achieving universal health coverage and leaving no one behind.

## Introduction

In many countries worldwide, there is a significant concern regarding inadequate sexual and reproductive health (SRH) among adolescents and young people [1]. This population group faces an elevated risk of acquiring Sexually Transmitted Infections (STIs) such as Human Immuno-Deficiency Virus (HIV), as well as experiencing violence and unintended pregnancies, particularly among young girls [1]. Additionally, adolescents and young people encounter various inequalities such as limited access to information, discrimination, exclusion, and violence [2–4]. Given that approximately 1.8 billion individuals, representing a substantial portion of the global population, fall within the age range of 10 to 24 years, addressing SRH risks is a matter of global health concern [5].

In low and middle-income countries (LMICs), adolescents and young adults aged 15–24 account for approximately one-fifth of the population [6]. However, the needs of this population, especially regarding sexual and reproductive health, are often unmet, overlooked, and underfunded [7, 8]. The challenge of sexual and reproductive health risks is a concern particularly, in Sub-Saharan Africa where its young people continue to grow substantially [6]. Zambia, in line with the region, faces similar challenges regarding sexual and reproductive health risks, with 63.2% of the country’s population being under 25 years old [9].

There is a growing demand on governments and key stakeholders to establish an enabling environment that empowers the younger population to fulfil their potential, which includes upholding their basic human rights and ensuring access to high-quality sexual and reproductive health (SRH) services [4, 10]. Studies conducted in various settings indicate a lack of utilization of services among adolescents and young people, particularly in the areas of mental health and SRH services [11, 12]. Despite governmental efforts to enhance access to SRH services, adolescents and young people continue to encounter challenges in meeting their specific health needs. Common obstacles include insufficient knowledge about adolescent sexual behaviour, cultural influences, limited access to reproductive health information, and the absence of adolescent and youth-friendly SRH services [13–15]. According to the Zambia Demographic Health Survey (ZDHS) conducted in 2018, only 43% of young women and 41% of young men have comprehensive knowledge of HIV prevention [16].

University students are particularly vulnerable to the impact of risky sexual behaviour and experimentation [17]. The increasing presence of public and private Higher and Tertiary Education Institutions (HTEIs) in Zambia has expanded access to education, making HTEIs crucial settings for service delivery and information dissemination in the field of Sexual and Reproductive Health and Rights (SRHR) through youth-friendly health services [18]. Adolescent Youth-Friendly Health Services (AYFHS) aim to address the barriers that young people face in accessing SRH services, and these services are expected to meet established international guidelines and standards for youth-friendly services [19]. However, there is limited evidence regarding the availability, accessibility, acceptability, and quality of SRH services in HTEIs in Zambia.

Current sustainable strategies for creating change are shifting towards human rights-based approaches that prioritise rights over needs [20]. The Right to Health approach encompasses the elements of Availability, Accessibility, Acceptability, and Quality (AAAQ) [21], which serve as a framework for identifying barriers throughout the entire treatment process. Availability ensures the presence of functioning health services in sufficient quantities, while accessibility guarantees non-discriminatory, physically and economically accessible services, along with accessible information. Acceptability entails that health services adhere to medical ethics, cultural appropriateness, gender and age sensitivity, and that medical treatments are explained in understandable ways. Quality necessitates that health facilities and medicines are scientifically and medically appropriate and of high quality [22, 23].

Applying the AAAQ criteria is crucial for understanding how young people exercise their right to healthcare when seeking and utilising services in higher education institutions. However, there is limited evidence regarding the perceptions of adolescents and young people regarding youth-friendly SRH services using the Human Rights Based Approach to Health Care and AAAQ framework in selected HTEIs. Therefore, this study was conducted to explore the perceptions of adolescents and young people concerning youth-friendly SRH services using the Human Rights Based Approach to Health Care and AAAQ framework in selected colleges and universities in Zambia.

## Methods

### Study setting

The study encompassed twelve (12) Higher and Tertiary Education Institutions (HTEIs) in Zambia, namely Evelyn Hone College (Lusaka Province), Chalimbana University (Lusaka Province), Lusaka Business and Technical College (Lusaka Province), Mulungushi University (Central Province), Kabwe Institute of Technology (Central Province), University of Zambia (Lusaka Province), Kwame Nkrumah University (Central Province), Lusaka Business and Technical College (Lusaka Province), Copperbelt University (Copperbelt Province), Nkumbi International College (Central Province), Technical Vocational Teachers College (Copperbelt Province), Northern Technical College (Copperbelt Province), and Mukuba University (Copperbelt Province). The selection of health facilities was based on students’ utilisation of health services. The chosen study sites are public institutions that host majority of young people. The sites encompassed diverse locations and sizes to enhance variation and representation.

### Study design

A qualitative case study design was employed to thoroughly investigate the viewpoints of adolescents and young people regarding youth-friendly health service delivery in Higher and Tertiary Education Institutions (HTEIs). Focus Group Discussions (FGDs) and In-Depth-Interviews (IDIs) were conducted with adolescents and young people and health care providers, while Key Informant Interviews (KIIs) were conducted with district level and provincial level AYFHS coordinators. This approach allowed for an exploration of the three key domains of the Rights-Based Framework: Availability, Acceptability, and Accessibility, while KIIs were specifically employed to delve into the domain of Quality.

### Sampling and recruitment of participants

Purposive sampling was employed to select participants for the study, ensuring a deliberate and targeted approach. Through institutional records and with permission from authorities, young people who were available were identified and invited to take part in the study. Additionally, interviews were conducted with healthcare facility staff and AYFHS coordinators at district and provincial levels to enhance the credibility of the findings. Snowball sampling was utilised to select young individuals living with HIV and from the LGBTIQ community, taking into consideration their potentially undisclosed identities, in order to explore their knowledge and utilisation of services in-depth. This approach aimed to enhance the validity of the findings without solely relying on increasing the sample size until theoretical saturation was achieved. Adolescents and young people of age group 18 and 24 years were included in the study. All individuals who refused to sign the informed consent were excluded from the study. A total of 112 interviews were conducted. Table 1 provides a comprehensive summary of the interviews conducted and the list of participants targeted for the qualitative study.

**Table 1:** List of participants interviewed.

Type of participants	Approach	Respondents	Number of discussions	No of participants per session	Total number of participants
Adolescent Health coordinators	KIIs	National, provincial and district level health directors	4	4	4
Health care providers	KIIs	Health facility in-charge	12 (One at each facility)	1	12
		Health care providers for youth friendly services	12 (One at each facility)	1	12
Young people,	IDIs	5 in each institution (1 with disabilities, 1 with HIV, 1 with LGBTIQ, 1 male and 1 female)	60 (5 in each institution)	1	60
Young people,	FGDs	2 in each institution (1 with females and 1 with males)	24 (2 from each institution)	6 per FDG	144
Total interviews			112		232

### Data collection methods

FGDs, KIIs, and IDIs were carried out during a three-month period from March to June 2021. The research team developed open-ended interview guides for respective participants after reviewing existing literature and consulting with the local research partner and the Ministry of Health. Information sheets were provided to participants prior to the guided discussions. Face-to-face KIIs, IDIs, and FGDs were conducted, and data collection involved the use of digital voice recorders and field note taking. To adhere to social distancing measures due to COVID-19, each discussion involved a maximum of six participants. Research assistants were trained in effective data collection methods and the discussions took place at agreed-upon private locations such as in the hostels. FGDs lasted for 60 minutes with up to six participants, while IDIs and KIIs lasted about 30 to 40 minutes each.

### Data management and analysis

The audio recordings of interviews were transcribed verbatim by two trained research assistants. The transcribed documents in Microsoft Word were carefully reviewed and analysed to develop a coding scheme. In vivo coding was applied, and two independent coders coded each transcript to enhance the reliability of interpretations. All transcripts were then imported into NVivo version 11, a qualitative software, for coding and ongoing analysis. The agreed-upon codes were organised to create categories and subsequently themes. A thematic analysis approach was employed to provide context to the findings of this study.

### Quality control

Research assistants underwent a two-day training session before the study commenced. The research tools were pretested to identify any potential deficiencies and to ensure participants’ understanding of the questions in the same way. The practice sessions and data collection were conducted in English, Chinyanja, and Chibemba languages. The fieldwork recruiters were also trained to screen participants and confirm their eligibility.

### Ethical considerations

Ethical approval for the study was obtained from the University of Zambia Biomedical Research Ethics Committee (UNZABREC—Ref-1588-2021). Permissions to collect information from health facilities were granted by the National Health Research Authority (NHRA). Additionally, permissions to access the facilities and engage with young people were granted by the Ministry of Health at the national, provincial, and district levels, as well as the administrative management at the tertiary institutional level.

### Findings

The aim of this study was to explore the perspectives of young people and healthcare providers regarding the accessibility, availability, acceptability, and quality of AYFHS in selected HTEIs in Zambia. The following section presents the viewpoints of young people and other stakeholders on these aspects of sexual and reproductive health services including youth friendly corners.

### Accessibility and barriers to accessing services

Based on the Human Rights-Based AAAQ framework, access to health services encompasses physical, economic, and information accessibility, while ensuring non-discrimination in service provision.

#### Physical access to services

In this study, some young people identified geographical access to public health facilities as a hurdle, especially when these facilities were not located within their educational institutions’ campuses. Students attending institutions without on-campus health facilities reported challenges in accessing adolescent and youth-friendly health services (AYFHS), as they had to travel to off-campus health facilities.

“We do not have a clinic on this campus, although we have clinics in the surrounding areas, it is not very easy to get there unless using a taxi or bus. Sometimes we walk though, but not at night” **[Participant 1, Group Discussion, Male, Lusaka]**

#### Access barriers for young persons with disabilities

Young persons with disabilities faced specific challenges in accessing campus health facilities because of the architectural designs which did not favour them, as well as the issue of queuing for services. The existing facilities were not designed to accommodate their needs, making it difficult for them to navigate and access the services. Moreover, the processes within the health facilities were not inclusive and posed additional difficulties for young people with disabilities. These individuals expressed their frustration with the lack of efforts to make the facilities more accessible and inclusive for them.

(…) we have different challenges, some of us…we are not able to stand for a long [period of time], and when you go there you will be forced [to] stand in the queue and in the process, you become tired (…) **[In-depth Interview, young person with a disability, Male-Kabwe].**

Some healthcare providers acknowledged the importance of addressing the accessibility needs of young persons with disabilities and made efforts to ensure that these students were served. These providers recognised the challenges faced by individuals with disabilities and expressed their commitment to making the necessary accommodation and adjustments to meet their needs. They were aware about accessibility challenges and the need for accessible services for students with disabilities. As such, efforts to ensure that services were accessible and convenient were being made. Their efforts demonstrated a variation in the level of responsiveness and inclusivity within the healthcare system towards young persons with disabilities.

“As for students with disabilities, we have those and we have tried as a facility to make it convenient and easy for them to access the services and we do certainly, we try by all means to prioritise them as they come just to make sure that they are served” [**Key-Informant Interview, Healthcare Provider].**

#### Affordability of services

Young people highlighted the issue of economic accessibility when accessing sexual and reproductive health services. They mentioned that they could only access services once they had paid their school fees, indicating a financial barrier to accessing healthcare. While certain medications like emergency contraception were available illicitly on campuses, other services could only be obtained within the health facility premises, where charges were sometimes imposed on the students. This financial burden compromised the economic accessibility of services. Additionally, some health facilities charged students for services during vacation periods, further exacerbating the economic challenges faced by young people in accessing necessary healthcare.

“Like I mentioned, the challenge is that you cannot always get the services we are talking about unless you are a paid-up student. Otherwise, when school closes you have to pay to get the same services” **[Participant 3, Group Discussion, Male- Lusaka].**

The study findings revealed significant challenges in the availability of essential drugs and commodities for sexual and reproductive health services. Young people reported that drugs like fungal treatment, emergency contraception, and condoms were either not available or frequently unavailable at the health facilities on campus. As a result, they had to purchase these drugs from other sources, increasing their healthcare expenses. This situation had further implications as students had to allocate their limited financial resources to healthcare instead of other important educational needs. The study also uncovered the emergence of illicit on-campus sale of medication, as indicated by a poster advertising the sale of the "morning-after pill" in one of the hostels. Health care providers recognized challenges resulting in reduced access to services for young people. When asked what would make it easy for them to provide these services, one healthcare provider reported logistical challenges, and stated the following.

“Our biggest challenge here is staffing [and] drug shortages such that most times we are not able to meet the needs of these young people at the facility” **[Key-Informant Interview, Healthcare Provider].**

#### Access to information

Limitations in the access to information about available services for young people were highlighted. Participants mentioned that their knowledge and use of services was restricted to those they were aware of. Commonly mentioned services included contraception (including condoms), STI screening, HIV testing, and pregnancy testing. It was observed that young people primarily relied on information shared by their peers to learn about these services. This indicates a lack of comprehensive and targeted information dissemination regarding the full range of services available at the institutions. A respondent expressed their experience, stating.

“For me I think, what I can say is that, we hear about these services from fellow students and also from the health chairperson who is also a student, that is all…really” **[Participant 6, Group Discussion, Female- Lusaka].**

#### Discriminatory attitudes as a barrier to accessing services

The study findings shed light on the presence of discriminatory attitudes among some healthcare providers, leading to barriers in accessing services. Young people reported encountering poor attitudes and unpleasant experiences when seeking healthcare. Some healthcare providers were unavailable when needed, while others exhibited negative attitudes towards young people seeking certain services. The young people expressed feelings of discrimination, paternalistic control, and stigmatisation, indicating a lack of understanding and support from healthcare providers. Instances were mentioned where young people were ridiculed or admonished for seeking information on sensitive topics such as safe abortion and access to condoms. One participant shared their experience, stating:

“We come to school leaving our parents’ home. But then when we come here [to the health facility], we find that in the health facilities, there are nurses who want to treat us like [we are] their children, by telling us why should we be having sex when we are here to learn” **[Participant 4, Group Discussion, Male, Lusaka].**

As a result, many young people preferred to avoid healthcare facilities unless they were seriously ill while others felt more comfortable seeking information from their peers or avoiding seeking information altogether unless they were very sick.

The study findings highlighted the particularly challenging situation faced by the LGBTIQ populations regarding access to information and services. It was observed that there was a lack of specific information tailored to their needs, indicating a bias in service provision. When asked about the availability of services that meet their needs, an LGBTIQ young person shared their perspective, stating:

“No, I don’t think they would even talk about that [tailoring LGBTIQ services], they wouldn’t even bring it up, I know, they wouldn’t just bring it up, I’ve been in this institution for quite a long time (…)” **[In-depth Interview- LGBTIQ, Female, Ndola].**

### Availability

According to the AAAQ framework, availability of services refers to the presence of public health and healthcare facilities in sufficient quantity, considering the developmental and economic conditions of a country. Self-reported information on service availability was obtained from both health care providers and young persons. We have found that services were available, but they were primarily focused on curative health services and not specifically tailored to the needs of adolescents and young people. Furthermore, the availability of services varied across different facilities. All the health facilities included in the study were open seven days a week, but most of them provided services only during regular working hours, from 8 am to 5 pm.

#### Available services

The study identified a range of services that were reported as available in the selected higher education and tertiary facilities. These services included voluntary HIV testing services, contraception, safe motherhood, cancer screening, mental health services (including for alcohol abuse), safe abortion services, sexual and gender-based violence awareness, management of chronic conditions, and discussions related to sex and sexuality. These services reflect the efforts made to address various aspects of sexual and reproductive health as well as general health concerns among the young population.

#### Unavailable services

The study findings revealed that certain services were consistently reported as unavailable in some of the higher education institutions visited. These included mental health services, contraception (including condoms), male circumcision, cervical cancer screening, safe delivery services, and treatment for yeast and fungal infections. Among these, mental health services were mentioned most frequently as being unavailable. Additionally, young people expressed a need for more comprehensive laboratory services, particularly for STI screening. The absence of abortion-related counselling, information, options, and services was also noted as a significant gap. These findings highlight the limitations and deficiencies in the availability of essential healthcare services for young people in the studied institutions.

“Services around abortion or even counselling, those are not provided here, maybe unless University Teaching Hospital” **[Participant 5, Group Discussion, Female].**

A key informant also confirmed non-provision of abortion services at one of the biggest institutions, saying:

“I think most preventive (…) services are the ones that we provide. And also, curative [services] if somebody is sick, we do that yes. But services like uh like you mentioned maybe providing the post-abortal… care or whatever, usually we don’t provide such services to those institutions.” **[Key Informant-District Adolescent health coordinator]**

#### Availability of awareness programmes

According to the perspectives shared by students, there is a perceived need for improved availability of Post Exposure Prophylaxis (PEP) and Pre-Exposure Prophylaxis (PrEP) services. These services were reported to be available only at one specific health facility within the institutions, indicating limited access for students in other locations. Furthermore, some students mentioned they had not accessed a wide range of services, which affected their awareness and knowledge of the available services in the health facilities. Therefore, there is a call for increased availability and accessibility of PEP, PrEP, and other services to better meet the needs of the student population. When asked what services he knew about, a young man from an institution in Lusaka said this:

“It would be good to know the services that are available for us. Unfortunately, we hardly know these services including HIV prevention medication, those are important…. personally, I did not know that HIV prevention medication were available until most recently.….” **[Participant 2, Group Discussion, Male].**

#### The role of guidelines in ensuring availability of services

The absence of guidelines governing the provision of Adolescent and Youth-Friendly Health Services (AYFHS) in the institutions was identified as a factor contributing to the limited availability of services. This lack of guidelines had a direct impact on the provision of services, even in the presence of healthcare providers within the facilities. A health facility in-charge expressed the belief that the availability of guidelines would enhance the provision of services and improve their availability. This highlights the importance of having clear guidelines in place to ensure the availability and effective delivery of AYFHS.

“We do not have any [guidelines]. We are just using the information we got from school [during training, but no specialised training]. When it comes to orientation of new guidelines, policies, we are not involved.” **[Key-Informant Interview, Health Facility In-charge].**

#### Regular shortages of drugs

A common complaint among the young people interviewed was the limited availability of medical supplies and specialised services in the health facilities. It was often mentioned that only basic medications like paracetamol (Panadol) were consistently available, while other essential services such as STI screening, cervical cancer screening, and safe abortion were not accessible due to the unavailability of equipment in the facilities. Frequent stock outs and the general unavailability of medical supplies were also highlighted by the young people. A student from a college in the Copperbelt Province shared their experience, emphasising the unavailability of necessary services.

But as a student, you are in your last days [you are broke], you do not have money, where are you going to get that money to buy medicine? So obviously you just stay in your hostels, you do not even go to class until you are fine. And for the clinic, that side, at least they have even upgraded [provide more services], and they will give you Panadol (…)” **[Participant 1, Group Discussion, Female, Copperbelt].**

Notably, the drugs for most key populations, particularly young people who identified themselves as LGBTIQ+, were not available to meet their specific needs. Almost all the facilities lacked specific services for the LGBTIQ+ community. Healthcare providers acknowledged this gap and expressed their views on the provision of LGBTIQ+-tailored health services including commodities. Their statements highlighted the need for inclusive and non-discriminatory care for LGBTIQ+ individuals.

“For LGTBQ+s, those we don’t really offer services but we just give them information on maybe how these people can be linked to support groups” [**Key-Informant Interview, Healthcare Provider**].

“The LGBTIQ community, ah! For those, I must be honest to say that we don’t have, as a facility, we don’t have a specific policy that helps us to address their needs comprehensively” **[Key-Informant Interview, Health Facility In-charge].**

Healthcare providers in all the facilities acknowledged the general availability of Adolescent and Youth-Friendly Health Services (AYFHS). However, they recognised that the available services were not specifically tailored to meet the needs of young people in HTEIs, including those with special needs. While health service provision for students living with HIV was reported to be available, there were only a few facilities that made efforts to adequately cater to the needs of students with disabilities. A healthcare provider highlighted the importance of providing services that are inclusive and accessible for students with disabilities.

“As for students with disabilities, we have tried as a facility to make it convenient and easy for them to access the services and we do certainly, we try by all means to prioritise them as they come just to make sure that they are served” **[Key-Informant Interview, Healthcare Provider]**.

However, despite the challenges reported, interviewees mentioned that additional support in the form of funding from NGOs that support youth-friendly corners made it easier for them to provide services to young people. Healthcare providers also highlighted specific aspects that would enable them to better provide these services. A health facility in-charge emphasised the importance of adequate resources and training for healthcare staff to effectively address the needs of young people.

“We do receive funds for SRH although it is not always, or should I say not consistent and this same support comes in form of ‘imprest’, although other partners also occasionally support us” **[Key-Informant Interview, Health facility In-charge].**

In confirming whether there was a budget for Youth-Friendly Services (YFS) that supported district-level programmes, a Key Informant (KI) stated that not much is happening with funding for such programmes. The KI highlighted the lack of sufficient financial resources allocated to support YFS initiatives at the district level.

“Most of the time actually [the] adolescent has been left behind, in terms of the budget and also allocation of the monies. Because um… the only people that have actually now come on board to help us are our partners. But where the government is concerned, they are not supporting us much”. [**Key informant- District Adolescent Health Coordinator]**

### Acceptability

For health services to be considered acceptable, they need to be ethically and culturally appropriate, demonstrating respect towards individuals, minorities, communities, and being sensitive to gender and life-cycle requirements. Young people in the study highlighted that the acceptability of certain services varied. They mentioned that services such as malaria screening were more acceptable compared to services related to sexual and reproductive health and rights (SRHR), including contraception and safe abortion. This difference in acceptability was particularly evident in a Christian mission facility that did not provide contraception and safe abortion services. The perceived unacceptability of certain services hindered young people from accessing them. One student expressed their frustration with this situation.

Contraceptives… I hear they are there [available] but they [healthcare providers] are judgemental. You know, at that point, that’s where (…) there’s a need of confidentiality and such services and I think… people are complaining. I have heard about that**. [In-depth Interview, Male- Ndola].**

However, it was observed that healthcare providers who were more supportive and welcoming towards young people had a positive impact on the acceptability of services. Their attitudes and behaviour were associated with increased demand and utilisation of services among students. Conversely, some health facility staff were perceived as unapproachable, with age differences being cited as a barrier to the acceptability of services. A young person from a higher education and tertiary institution (HTEI) in Lusaka shared their experience regarding this matter.

(…) the issue of having somebody who is in a different generation from yours because when you want… When we want to access services as young people, we hope to be in a safe space where [we can be] free or open and honest. But when you find somebody who’s in a different generation from yours or a different age range, it’s hard for you to communicate effectively because you would think they won’t understand you and you think they’re going to come at you in a judgmental way (…) **[In-depth Interview, Female- Lusaka].**

Young people participating in the study highlighted that health facilities within their institutions were perceived as more "user-friendly" compared to external facilities. The presence of a health facility or youth-friendly space within the learning institution was seen as favourable by young people, as it increased the acceptability of using the services and facilitated access to AYFH services. Additionally, the role of peer educators was highly valued. In most institutions, peer educators were considered knowledgeable and easily accessible for information and services like condom distribution. One student expressed their appreciation for peer educators in the following statement.

“For me I find peer educators to be helpful, you know for us young people, it is easy to joke with these people and I think they also understand what we as young people go through” **[In-depth Interview, Male-Kabwe].**

### Quality

The interviews conducted highlighted concerns regarding the quality of services provided in these institutions. Across most health facilities, issues such as inadequate supplies, insufficient space for privacy, lack of necessary equipment, and insufficient training to meet the specific needs of students were mentioned. A healthcare provider acknowledged the poor quality of services, attributing it to underfunding of programmes. This sentiment was expressed by one healthcare provider.

“Our facility is always rated in red when we get assessed” **[Key-Informant Interview, Healthcare Provider].**

#### Lack of, and inadequate youth-friendly spaces

During the individual interviews with healthcare providers, it was revealed that the lack of, and inadequate youth-friendly spaces posed significant barriers to providing quality services. The quality of services was compromised in facilities with insufficient infrastructure or limited space for ensuring privacy. Many young people expressed concerns about the inadequacy of the spaces provided, citing them as either too small or lacking sufficient privacy. These concerns were further amplified by the ongoing COVID-19 pandemic, as open and crowded spaces were seen as potentially dangerous.

I wanted to talk about it [inadequate space], it’s not spacious there and there is congestion. Especially with this pandemic, so it’s not that healthy because it’s too small. **[Participant 5, Group Discussion, Female, Copperbelt].**

In contrast, in some institutions, youth-friendly spaces were completely non-existent, as reported by young people. A participant from a university expressed their dissatisfaction with the lack of dedicated spaces for health service provision, especially considering the growing student population. They highlighted the need for more private locations to accommodate the increasing demand for services. Healthcare providers supported this viewpoint, acknowledging the shortage of youth-friendly spaces, particularly during the rainy season when the open areas were more challenging to utilise effectively. A healthcare provider stated their concerns regarding the limited availability of appropriate spaces for providing quality services.

“Youth-friendly spaces are a challenge because sometimes in the rainy season, the young people have nowhere to go” **[Key-Informant Interview, Health Facility In-charge].**

Due to the constraints of limited space, healthcare providers reported that services were scheduled on specific days and could not be provided continuously. This restricted the availability and accessibility of services to young people. In addition, a healthcare provider highlighted the inadequacy of their youth-friendly corner, describing it as very small and lacking chairs for young people to sit. This further compromised quality service delivery and the overall experience for the young individuals seeking care.

“The spaces for AYP are small such that sometimes health education is provided from outside” [**Key-Informant Interview, Health Facility In-charge].**

During the interviews, it was revealed that healthcare providers in the institutions running health facilities separate from the Ministry of Health (MoH) lacked professional training in Adolescent and Youth-Friendly Health Services (AYFHS). When asked about their training in AYFHS, one interviewee expressed the following:

“I have not had any training [specialised AYFHS training]” **[Key-Informant Interview, Healthcare Provider].**

This lack of specialised training further contributed to the barriers and challenges faced in delivering high-quality health services to young individuals in these institutions.

Young people in institutions that rely on regular health facilities outside their institutions expressed challenges with long waiting hours to access Adolescent and Youth-Friendly Health Services (AYFHS). They highlighted the need for more tailored services that specifically cater to their needs, as they did not appreciate the long queues in general health facilities. Accessing these services often required waiting in queues with other patients, which discouraged them from seeking the services altogether. The long waiting hours were seen as a barrier to accessing timely and efficient healthcare, and young people felt that their specific needs were not being adequately addressed in the crowded general health facilities. As a result, they expressed a desire for more targeted and youth-friendly services that prioritise their unique requirements and ensure a more streamlined and efficient healthcare experience.

“Sometimes you may not even access the services because [when] people get to the clinic, [they/we] have to queue up, so the thought is… I wish there was a place where we just go as students and get a service” **[Participant 1, Group Discussion, Male- Lusaka].**

A healthcare provider in charge of AYFHS also mentioned that she had no professional training, despite accepting the appointment as the facility’s AYFHS nurse. This highlights the lack of specific training and expertise in providing tailored healthcare services for young people. Without proper training, healthcare providers may struggle to understand the unique needs and challenges faced by young individuals and may not be equipped with the necessary skills to deliver quality AYFHS. The provider’s statement reflects the importance of investing in professional training programmes for healthcare providers who are involved in delivering AYFHS. Such training would help them acquire the knowledge and skills needed to effectively address the physical, mental, and reproductive health needs of young people. By enhancing their training, healthcare providers can better understand the principles of youth-friendly care, develop appropriate communication strategies, and create a supportive and non-judgmental environment for young patients seeking healthcare services.

*“*Previously what I got (is) from the handovers they had [was too basic]. However, most of those who were in that group [at] that time, they have left. But right now, I don’t have any training. I was just told to carry on, “*we will train you when the time comes*” [**Key-Informant Interview, Healthcare Provider**].

The key informants emphasised the need for training more AYFHS care providers, as many of them tend to be transferred to different facilities or pursue further training. This highlights the issue of staff turnover and the potential impact it has on the continuity and quality of AYFHS. Without a sufficient number of trained providers, the delivery of youth-friendly services may be compromised. One key informant added;

Human resource, there is a gap in terms of training. People get trained but with time some get transferred, some die and some retire. We had a lot who were trained but after the 2015/2016 training, there has not been any training and we have a high attrition rate. People retired, others got transferred and others went to school, they are on long study leave” **[Key-Informant Interview, District Adolescent Health Coordinator].**

The interviews with stakeholders revealed several administrative challenges in the management of facilities providing services for young people. These challenges included insufficient funding, inadequate monitoring, and the absence of specific guidelines to ensure the provision of services at the appropriate quality. The lack of guidelines resulted in variations in the management of services across different education institutions, as the responsibility was shared between the health and education ministries. The inadequate funding posed a barrier to effectively meeting the needs of young people, including providing necessary resources, equipment, and training for healthcare providers. Insufficient monitoring mechanisms further hindered the ability to ensure the quality of services being delivered.

Despite these challenges, stakeholders confirmed that there is strong collaboration between the Ministry of Education and the Ministry of Health in supporting the health facilities. This collaboration involves technical support, such as hiring and training of healthcare providers, as well as supervisory visits to ensure the provision of quality services. Addressing the administrative challenges requires increased funding allocation, development of specific guidelines for AYFHS management, and strengthening monitoring and evaluation mechanisms. By addressing these issues, there can be improved coordination, resource allocation, and overall management of health facilities, leading to better quality AYFHS for young people. In addition, funding for the facilities was dependent on the entity in charge of the management of the facility.

“The health facility is owned by [the university] and not by the Ministry of Health, rendering it to be a private health facility. However, this health facility is generously supported by the Ministry of Health and its partners” **[Key-Informant Interview, Health Facility In-charge].**

District level management recognised the limitations in funding for AYFHS in health facilities, including those in higher learning institutions. In facilities run by MoH, funding primarily comes from MoH through district-level grants. However, these facilities also receive support from educational institutions. On the other hand, facilities run by universities or colleges largely fund their operations through medical fees paid by students and support from the MoH. The statements suggest that funding for AYFHS in these health facilities is a shared responsibility between the MoH and educational institutions.

However, it is acknowledged that there are limitations in the available funding, which can impact the provision of comprehensive and quality services for young people. To address these funding limitations, it may be necessary to explore additional funding sources, advocate for increased financial support from relevant stakeholders, and prioritise allocation of funds specifically for AYFHS. This can help ensure sustainable funding for the provision of youth-friendly services in health facilities, including those in higher learning institutions.

“Like I said the major source of funding is through student’s fees that these students contribute among the other requirements, which is medical fee. So that is the money that we use to buy drugs. Although certain times we actually use any amount that is generated by management” **[Key-Informant Interview, Health Facility In-charge].**

At the policy implementation level, the absence of guidelines resulted in the delivery of services for young people being similar to those provided to the general population. Health facility management and healthcare providers in higher learning institutions, both under the MoH and the Ministry of Education (MoE), were unaware of existing guidelines for the provision of AYFHS. There was a lack of knowledge about the current strategic plan being implemented, and many providers mentioned a lack of training and dissemination of these documents. District AYFHS coordinators also acknowledged a gap in information sharing regarding AYFHS guidelines, policies, and programme implementation between the MoH and higher learning institutions.

## Discussion

The findings from this study highlight notable challenges in terms of accessibility, availability, acceptability, and quality of sexual and reproductive health services for young people in health facilities within HTEIs in Zambia. These barriers are discussed, within the context of other literature.

While basic services were available, our study revealed that geographical, commodity-related, and human resource-related barriers hindered access to youth-friendly healthcare services. Geographical barriers were identified as a challenge, as some institutions lacked on-site health facilities offering youth-friendly services. This necessitated young people to travel considerable distances to access healthcare services, including sexual and reproductive health services. This finding aligns with a study conducted by Pandey et al. [24], which identified distance to the nearest health facility as a major barrier to the utilisation of adolescent and youth-friendly health services (AYFHS).

Inadequate infrastructure with limited privacy was identified as another significant barrier to accessibility. Adolescents expressed hesitancy in accessing sexual and reproductive healthcare services due to concerns about their privacy being compromised. They feared that others would overhear or become aware of their personal health conditions. This finding aligns with the research conducted by Pandey et al. [24], which highlighted the lack of privacy and confidentiality as a major deterrent for adolescents seeking to utilise adolescent and youth-friendly health services (AYFHS).

Young people expressed dissatisfaction with the unacceptability of healthcare services provided at most learning institutions. They reported shortages, basic care, and a lack of healthcare providers who understood their specific needs. Negative experiences arising from poor attitudes displayed by healthcare providers further hindered adolescents’ access to youth-friendly health services. Discrimination, paternalistic control, and stigmatisation were cited as major reasons for not seeking health services, as healthcare professionals exhibited these behaviours. This finding aligns with a study by Tilahun et al. [25], which found that one-third of healthcare workers held negative attitudes toward offering reproductive health services to unmarried adolescents.

Services in the health facilities were not adequately tailored to meet the needs of key populations, such as the LGBTIQ community and persons with disabilities. These groups faced challenges in accessing services and information, primarily due to lack of accommodation, privacy, and understanding from healthcare professionals. Adolescents belonging to key populations, particularly those identifying as LGBTIQ, reported encountering negative attitudes and biases from healthcare providers, which deterred them from utilising the facilities for sexual reproductive health services (SRHS) [26]. A study conducted by Müller et al. [27] revealed that although health facilities did offer adolescent sexual and reproductive health services (ASRH), they were not inclusive or accessible to sexual and gender minority adolescents.

In the institutions assessed, there were some general sexual and reproductive health (SRH) services available, but young people had limited awareness of the specific services offered. Their knowledge about reproductive health services was lacking, which affected their access to SRH services. Our study found that young people were mostly aware of, and commonly used a few services such as contraception, STI screening, HIV testing, pregnancy testing, and obtained information on these services from their peers. This lack of awareness can be attributed to the absence of health promotion and education activities conducted by healthcare professionals within the institutions.

Cultural, religious, and traditional norms played a significant role in hindering open discussions about sex and sexual matters [28]. The lack of trust in healthcare providers (HCPs) also limited access to SRH services among adolescents and young people. Our study revealed that even when individuals sought knowledge about SRH without being sick, they experienced harsh treatment from Health Care Providers (HCPs), which further limited their access. Similar findings have been noted by Lutende, who emphasised that harsh treatment by HCPs can result in a lack of competence and hinder adolescents’ access to healthcare services [29]. However, peer educators were recognised as valuable in increasing accessibility and acceptability of services. Overall, the limited awareness of available services, cultural barriers, and negative experiences with healthcare providers affected the accessibility and acceptability of SRH services for adolescents and young people.

Institutional barriers pose challenges for adolescents in accessing healthcare services, as highlighted in this study. Pandey et al. [24] conducted a study that identified staff shortages, inadequate SRH supplies, and a lack of medicine in health facilities as institutional barriers affecting the availability and quality of healthcare.

Consistent with our findings, although Adolescent and Youth-Friendly Health Services (AYFHS) were generally available as per policy specifications, certain services and specifically trained healthcare providers for those services were unavailable. Additionally, there was a lack of essential drugs needed by adolescents seeking healthcare. Furthermore, most of the services provided were not tailored to meet the specific needs of adolescents and young people, which had an impact on the overall quality of AYFHS. These institutional barriers contribute to compromised quality in AYFHS. It is important to address these barriers to ensure that healthcare services are accessible, available, and of high quality for adolescents and young people [24].

### Limitations

A limitation of this study was the ethical concerns associated with identifying key populations and respecting privacy and confidentiality. To address this, snowball sampling was employed to identify key-population participants. Another limitation was the inclusion of only 12 out of 62 Higher and Tertiary Education institutions in Zambia, which prevented achieving theoretical saturation. Further exploration of contextually driven reasons for young people’s choice of care provider could be pursued. However, the study attempted to increase the transferability of findings by including institutions that varied in type and location, reflecting the socio-economic and cultural landscape of the country [30]. Additionally, the credibility of the findings was enhanced by utilising diverse data sources and employing well-known methodologies for data collection. Transparent explanations of the data collection and interpretation processes were provided, increasing the overall trustworthiness of the research findings [31].

## Conclusion

The study findings indicate that although some Adolescent and Youth-Friendly Health Services (AYFHS) were available in HTEIs, only a few services were perceived as accessible by young people. This was due to factors such as limited opening hours, high costs, long waiting times, frequent shortages of equipment and commodities, and poor attitudes of healthcare providers. These findings suggest the need to improve programming for adolescents and young people in HTEIs by ensuring that services are readily available when needed and addressing the specific vulnerabilities of students with disabilities and key populations to achieve universal health coverage.

It is crucial to prioritise funding to support a systems approach in strengthening AYFHS in health facilities within HTEIs in Zambia. There is an urgent need to address challenges in service availability and expand the range of services offered to meet the health needs of adolescents and young people. Enhancing the competencies and skills of healthcare providers in delivering AYFHS, including reaching out to minority populations, can greatly improve service uptake and health outcomes among this population. Ensuring the availability of medical supplies, providing appropriate and sufficient information, and addressing the identified issues can significantly enhance the quality of services offered.
